# Effectiveness of Peer-Delivered Trauma Treatment in a Rural Community: A Randomized Non-inferiority Trial

**DOI:** 10.1007/s10597-019-00443-3

**Published:** 2019-07-23

**Authors:** Annette S. Crisanti, Cristina Murray-Krezan, Jessica Reno, Cynthia Killough

**Affiliations:** 1 Department of Psychiatry and Behavioral Sciences, University of New Mexico, MSC 09 5030, Albuquerque, NM 87131 USA; 2 Department of Internal Medicine, University of New Mexico, Albuquerque, NM USA

**Keywords:** Peer-providers, Seeking safety, Trauma-specific treatment, Post-traumatic stress disorder, Randomized controlled trial

## Abstract

This randomized controlled non-inferiority trial explored the effectiveness of Seeking Safety (SS) delivered by peer providers compared to its delivery by licensed behavioral health clinicians. The study enrolled 291 adults with PTSD and/or substance use disorders. Data were collected at 3 and 6-months post start of treatment. With respect to long-term outcomes, at 6 months PTSD symptoms decreased by 5.1 points [95% CI (− 9.0, − 1.1)] and by 4.9 points [95% CI (− 8.6, − 1.1)] and coping skills increased by 5.5 points [95% CI (0.4, 10.6)] and by 5.6 points [95% CI (0.8, 10.4)], in the peer- and clinician-led groups, respectively. This study demonstrated non-inferiority of peer-delivered SS compared to clinician-delivered SS for reducing PTSD symptoms and similar outcomes for both groups with respect to coping skills. A confirmatory study on the effectiveness of peer-delivered trauma-specific services is warranted, especially given the potential for increasing access to such treatment in underserved rural communities.

**Electronic supplementary material:**

The online version of this article (10.1007/s10597-019-00443-3) contains supplementary material, which is available to authorized users.

## Introduction

Clinical and general population studies on the prevalence and consequences of trauma have generated enough evidence for us to know two things for certain. First, exposure to traumatic events is prevalent among persons with mental illness and substance use disorders (Cusack et al. 2004; Foa et al. 2007; Kessler et al. 2005; Mueser et al. 1998). Second, the negative impact of trauma is far-reaching as it impacts physical, mental, social, and economic well-being (Anda et al. 2006; Dube et al. 2001; Choi et al. 2017). Seeking Safety (SS) is an evidence-based cognitive behavioral treatment designed to help survivors with co-occurring trauma, posttraumatic stress disorder (PTSD), and substance use disorders (Najavits 2002). While no specific degree or experience level is required to provide SS, most of its evidence of effectiveness comes from studies that have used trained behavioral health clinicians including substance abuse or mental health counselors, social workers, and psychologists (Cohen and Hien 2006; Cook et al. 2006; Desai et al. 2008; Morrissey et al. 2005; Najavits and Hien 2013; Wolff et al. 2012).

Peer providers “use his or her lived experience of recovery from mental illness and/or addiction, plus skills learned in formal training, to deliver services in behavioral health settings” (Peer Providers/SAMHSA-HRSA n.d.). With an increase in their evidence base, peer providers are being employed more and more to address the behavioral health workforce shortage, especially in rural communities (Davidson et al. 2012). The benefits of peer-delivered services are well documented (Davidson et al. 2012; Fuhr et al. 2014; Lloyd-Evans et al. 2014; Mahlke et al. 2017; Miyamoto and Sono 2012; Reif et al. 2014; Repper and Carter 2011; Rogers et al. 2009; Sells et al. 2006); however, research on the effectiveness of peer-delivered trauma-specific treatment is limited. Two non-experimental studies of peer-led SS found positive results including improvements in trauma-related symptoms and coping skills, lower utilization of costly behavioral health services, and reduced inpatient readmission rates (Najavits et al. 2014; OPTUM n.d.). No randomized controlled trials (RCTs) of peer-led SS have been conducted. Among the various trauma-specific treatments, SS is a viable and safe choice for implementation by peer providers mostly because it is a present-focused approach addressing current coping skills, psychoeducation, and managing symptoms for better functioning. In addition, it is a manualized approach that requires minimal training and can be conducted in groups. The purpose of our study was to determine the effectiveness of SS led by peer-providers compared to its delivery by licensed behavioral health clinicians with respect to decreasing PTSD symptoms and increasing coping skills. Our hypothesis was that peer-led SS (PL-SS) would be as effective as clinician-led SS (CL-SS) in improving outcomes.

## Methods

Through funding from the Patient Centered Outcomes Research Institute (PCORI—CE-12-11-4484), we conducted a comparative effectiveness non-inferiority RCT in a rural county in a Southwestern state. This study was approved by the Institutional Review Board at the local University and registered with ClinicalTrials.gov (Registry Number NCT02081417).

### Recruitment, Eligibility, and Randomization

Participants were recruited between January 2014 and May 2016. The primary recruitment site was a peer-operated community-based recovery center but recruitment was expanded to a nearby residential treatment program for substance use disorders for 3 months to achieve the required sample size to be fully powered to test the study hypothesis. Eligibility was determined at intake by a licensed clinical mental health counselor through a structured clinical interview using the Mini International Neuropsychiatric Interview (Sheehan et al. 1998). Those determined eligible were randomized to PL-SS or CL-SS. Study inclusion criteria included 18 years of age or older and current PTSD and/or substance use disorder based on DSM-IV criteria, which is the target population for SS. Study exclusion criteria included a psychiatric hospitalization or suicide attempt in the past 2 months and inability to provide informed consent to participate in the study. The self-report Life Events Checklist (LEC), that includes 16 events known to result in PTSD or distress, was also completed during the structured clinical interviews conducted at the recovery center only (Gray et al. 2004).

### Delivery of the Intervention

SS groups were gender specific. The male CL-SS group was facilitated by a male licensed Clinical Mental Health Counselor with an MA in Counselling. The female CL-SS group was facilitated by a female licensed Alcohol and Drug Abuse Counselor with an MS in Developmental Psychology. The male and female PL-SS groups were facilitated by peer providers (one male and one female) who were in recovery from a substance use disorder and certified in the delivery of peer support services through a state-level credentialing board for behavioral health professionals. All group facilitators were from the study catchment area and therefore representative of the target population. Peer providers and clinicians received the same level of training and supervision in SS. Twelve of the 25 SS topics were selected for implementation through a consensus process involving multiple and various stakeholders (list available online see Supplementary Material). This abbreviated implementation of SS is consistent with other studies (Anderson and Najavits 2014; Hien et al. 2015, 2009; Morgan-Lopez et al. 2014). The facilitators cycled through the 12 topics eleven times between January 2014 and June 2016 resulting in a total of 144 SS sessions during the study period. Groups were delivered once per week lasting 1.5 h. An open-enrollment group format was used which allowed participants to join a SS group as soon as they were determined eligible. Similar to other studies on SS, treatment completers were defined as those who attended six or more sessions (Ghee et al. 2009; Najavits et al. 1998). Sign-in sheets were used to track session attendance.

### Strategies to Encourage Retention

Several strategies were used to encourage treatment completion including the provision of light refreshments at all SS groups, a $10 gift card at the 6th and 12th session milestones, transportation to and from SS groups, weekly reminder and follow-up phone calls, and childcare services. In addition, motivational incentives using a fishbowl method based on an affordable contingency management approach were used during every session (Promoting Awareness of Motivational Incentives - NIDA/SAMHSA Blending Initiative Motivational Incentives Suite n.d.).

### Treatment Fidelity

Independent quarterly fidelity assessments were conducted by Treatment Innovations (a consultant firm developed by the creator of SS) using the SS Adherence Scale, Long Version (Najavits 2003). Group facilitators completed nine fidelity assessments throughout the study. The fidelity scale has a total of 21 items that are divided into three sections: format, content, and process. Items were rated on a scale ranging from 0 to 3, with a rating of 2 or higher indicative of high fidelity. The clinicians showed slightly higher fidelity to SS compared to the peer providers; however, all facilitators were implementing SS with high fidelity (Table available online see Supplementary Material).

### Outcome Measures

Individuals who completed at least one SS group participated in structured interviews immediately following their first group (referred to as the initial interview) and two follow-up interviews at 3 and 6 months. Participants received a $20.00 gift card for each interview. Interviews were completed by peer providers who were trained in data collection and not involved in the provision of any services. The primary outcomes were PTSD symptoms and coping skills. PTSD symptoms were assessed by the self-report 17-item Post-Traumatic Stress Disorder Checklist Civilian version (PCL-C) (Weathers et al. 1993). PCL-C scores range from 17 to 85 with higher scores reflecting higher severity. A change score of − 5 indicates treatment response, while a change score of − 10 is considered to be clinically meaningful (Monson et al. 2008). The Coping Scale developed for SS was used to assess the degree to which participants used 18 specific coping skills from SS (Gatz et al. 2007). Higher scores indicate greater frequency of use with the range of scores being 0 to 90.

### Statistical Analysis

The study implemented a non-inferiority design to determine whether PL-SS was as effective as CL-SS. Sample size estimates were based on the PCL-C because this measure was the most resistant to change and would contribute the most conservative (larger) estimate for the required sample size. With a minimum sample size of 64 in each arm, we aimed to achieve 80% power to detect a raw difference between changes over time of ≤ 2.5 points on the PCL-C between the study arms. After consultation with a faculty psychiatrist in the host institution’s Psychiatry Department and expert in PTSD, we determined that the change in PCL scores in both the PL-SS groups and CL-SS groups from baseline to post-intervention (i.e., 3-months), would be considered clinically non-significant if they fell within 2.5 points of each other with a standard deviation of 5. Per FDA guidance, standardized estimates should be used to control for variability in the raw differences in change scores (U.S. Department of Health and Human Services 2016). Therefore, the clinically hypothesized non-inferiority margin (NIM) is equivalent to a NIM = 2.84 in standard deviation units. Based on our hypothesis, non-inferiority could be declared if the 95% upper confidence limit (equivalent to a one-sided 97.5% CI interval typically reported for non-inferiority testing) for the standardized difference of change scores between arms was ≤ 2.84 standard deviation units. Previous results from the Coping Scale were not available at the time of study design so rather than hypothesizing a specific NIM for that outcome measure, we only report descriptive statistics. Demographics were collected on age, sex, ethnicity, race, education, living situation, and employment. Treatment completion was categorized as completing at least six sessions by 3-months, by 6-month follow-up, or never completing at least six sessions before the 6-month follow-up. To summarize traumatic experiences, three subscores were generated for the LEC including number of events (i) experienced, (ii) witnessed, and (iii) learned about. The events experienced and events witnessed were combined for a total number of types of lifetime traumatic events experienced or witnessed with scores ranging from 0 to 16.

Data were analyzed as intention-to-treat where all participants were analyzed with their assigned study arm. Due to the nature of this population, we expected considerable attrition and missing data (Crisanti et al. 2014). We assessed the missingness for systematic problems and for missing not at random and confirmed that, for each outcome, missingness was likely related to both observed and unobserved data, invalidating the use of multiple imputation to adjust for missingness. Instead, to minimize bias, we included other collected data in our models that could account for some of the variability due to the missingness: a dichotomous variable for missing any interviews versus missing no interviews, enrollment site, and living situation (house/apartment/group home/halfway house; homeless/shelter/friend or family’s home; prison/school/hospital). Also in mind was the goal to show that PL-SS was as effective as CL-SS and that missingness was not related to intervention arm.

Linear mixed models were fitted to each of the outcome measures including all time points to assess the effect of intervention group (PL-SS vs. CL-SS) over time and least squares mean estimates are reported in the Results. For all models, a first-order autoregressive covariance structure was assumed to account for observations closer in time being more likely to have a higher correlation than observations further apart in time. All models included intervention arm and covariates for age, gender, ethnicity (Hispanic vs. not Hispanic), completion of SS, missed interviews (any vs. none), site, living status, and time point (first interview, 3 months, and 6 months), as well as the interaction between intervention arm and time point. Assessing this interaction provided a test whether there were differential changes over time in the two treatment arms for PTSD and coping score outcomes. Additional two-way interactions were initially included in the full models between intervention group and each of gender and ethnicity; between gender and SS program completion; site and treatment arm; and between site and any missed interview, but none were found to be significantly associated with the outcomes so were removed from the models. Pseudo-residuals were assessed for normality and quality of model fitting. Mean estimates and changes reported in the results are least squares mean differences and 95% confidence intervals from the modeling. We report F statistics for fixed effect estimates from the models along with their numerator and denominator degrees of freedom (“ndf” and “ddf”, respectively) and corresponding p-values.

## Results

**Fig. 1:**
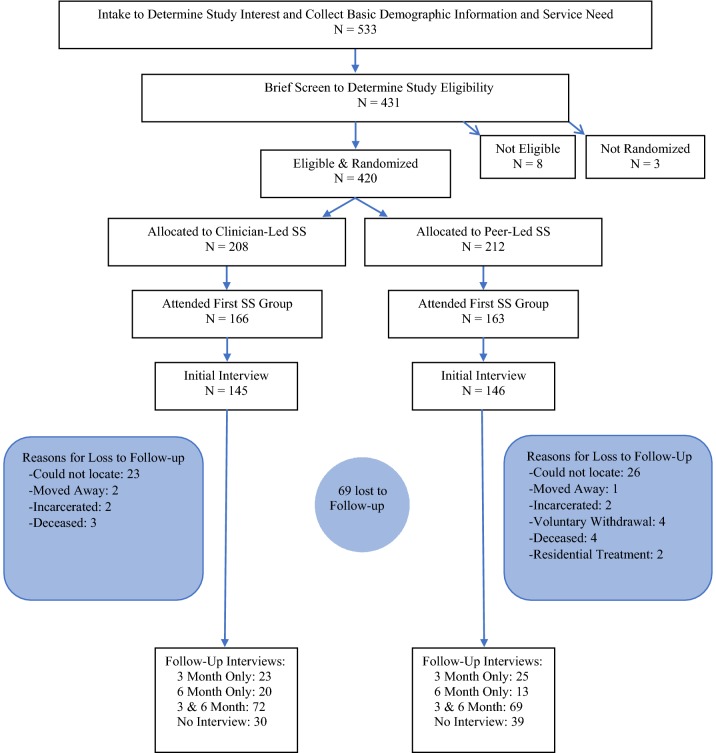
CONSORT diagram of participant flow

A total of 431 assessments for eligibility were conducted; 375 at the recovery center and 56 at the residential treatment program. Ninety-eight percent (n = 420) were determined eligible and randomized to a gender-specific SS group; CL-SS (n = 208) or PL-SS (n = 212). Of those determined eligible, 69% (n = 291) attended at least one SS group and consented to participate in the structured interviews: 145 in CL-SS and 146 in PL-SS. Of those who completed an initial interview, 67% (n = 222) participants completed at least one follow-up interview, with 48% (n = 141) completing both the 3 and 6-month follow-up interview. Sixty-nine participants (24%) were lost to follow-up (see Fig. 1). Enrolled participants (N = 291) were compared to those eligible, randomized individuals but not enrolled (n = 129) on several demographic and clinical variables and no significant differences were observed (Table available online). Demographic and clinical characteristics are summarized in Table 1. The mean and median number of SS sessions completed were similar for treatment arms: CL-SS was 5.7 and 4, respectively, and PL-SS was 6.4 and 5. By 3 months, 37% of participants in CL-SS and 38% of participants in PL-SS completed treatment (i.e., 6 SS sessions). By 6 months, the completion rate was 58% (CL-SS) and 54% (PL-SS).

**Table 1:** Demographics of participants by treatment arm

Characteristic	Treatment arm						p-Value^a^
	Clinician-led (n = 145)		Peer-led (n = 146)		Overall (N = 291)		
	Mean	Range	Mean	Range	Mean	Range	
Age, mean (range)	35	18–64	35	18–60	35	18–64	0.87
Traumatic events, mean (range)^b^	7	0–13	7	1–14	7	0–14	0.14

	N	%	N	%	N	%	
Gender, n (%)							0.59
Female	62	43	67	46	129	44	
Male	83	57	79	54	162	56	
Ethnicity, n (%)							0.73
Hispanic	121	83	124	85	245	84	
Non-Hispanic	24	17	22	15	46	16	
Race, n (%)							0.78
Caucasian	90	62	86	59	176	60	
Native American	16	11	17	12	33	11	
Asian	0	0	2	1	2	1	
African American	1	1	2	1	3	1	
Multiracial or other	38	26	39	27	77	26	
Employment, n (%)							0.50
Unemployed	116	80	112	77	228	78	
Employed	29	20	34	23	63	22	
Education, n (%)							0.14
4-year college or higher	6	4	6	4	12	4	
Some college	32	22	42	29	74	25	
High school Graduate/GED	59	41	41	28	100	34	
Some high school	43	30	46	32	89	31	
8th grade or less	5	3	11	8	16	5	
Living situation, n (%)							0.65
Apartment or house	78	54	89	61	167	57	
Halfway house/group home	7	5	5	3	12	4	
Hospital or detox center	2	1	0	0	2	1	
Jail/prison	12	8	14	10	26	9	
School or dorm	1	1	0	0	1	0	
Shelter/street	4	3	3	2	7	2	
Other	41	28	35	24	76	26	
Diagnosis, n (%)							0.50
PTSD only	14	10	12	8	26	9	
Substance use disorder only	34	23	43	29	77	26	
PTSD and substance use disorder	97	67	91	62	188	65	

^a^p-Values were calculated using a two sample *t* test for age and traumatic events; a Chi square test for gender, ethnicity, employment, education, and diagnosis; and a Fisher’s exact test for race and living situation

^b^Only available for participants recruited from the Recovery Center

PTSD symptoms decreased and coping skills increased similarly over time in both arms (intervention arm × time point interaction: F = 0.02, p = 0.98, ndf = 2, ddf = 357; F = 0.14, p = 0.87, ndf = 2, ddf = 355, respectively, Table 2). For both outcome measures, the differences between intervention arm change scores over time were not significantly different from each other and the 95% CIs overlapped considerably providing evidence that both intervention arms had similar reductions in PTSD symptoms and increases in coping skills over time (Table 3). The upper confidence limit (UCL) of the standardized 3-month—initial interview change score, the primary endpoint for PCL-C, was 2.1 which fell within the non-inferiority margin (UCL = 2.07 < NIM = 2.84). The main effect of intervention arm was not significantly associated with either outcome. Subjects in both arms reported lower PTSD symptoms and higher coping skills from the initial interview to 6 months (Table 2).

**Table 2:** Least squares mean estimates, standard errors (SE), and confidence intervals (CI) for outcome measures by time point and intervention arm, from models adjusted for independent variables of interest

	Overall			Clinician led		Peer led		Clinician led		Peer led	
	Estimated mean score	SE	95% CI	Estimated mean score	95% CI	Estimated mean score	95% CI	Change scores^a^ from initial	95% CI	Change scores^a^ from initial	95% CI
PCL total score				n = 145		n = 146		n = 145		n = 146	
Initial (n = 291)	47.7	1.7	44.3–51.1	48.2	44.3–52.1	47.2	43.3–51.1	–	–	–	–
3 months (n = 188)	44.3	1.8	40.8–47.8	44.7	40.5–48.8	43.9	39.7–48.1	− 3.5	− 10.8 to 4.8	− 3.3	− 7.0 to 0.4
6 months (n = 173)	42.7	1.7	39.2–46.3	43.3	39.2–47.5	42.1	37.8–46.4	− 4.9*	− 8.6 to − 1.1	− 5.1*	− 9.0 to − 1.1

	Overall			Clinician led		Peer led		Clinician led		Peer led	
	Estimated mean score	SE	95% CI	Estimated mean score	95% CI	Estimated mean score	95% CI	Change scores^a^ from initial	95% CI	Change scores^a^ from initial	95% CI
Coping total score				n = 145		n = 145		n = 145		n = 145	
Initial (n = 290)	46.1	1.6	42.9–49.3	46.5	42.8–50.2	45.7	41.9–49.4	–	–	–	–
3 months (n = 187)	48.4	1.8	45.0–51.9	48.3	44.2–52.4	48.6	44.5–52.7	1.8	− 3.0 to 6.6	2.9	− 7.9 to 7.7
6 months (n = 173)	51.6	1.8	48.1–55.2	52.1	47.9–56.2	51.2	46.8–55.5	5.6*	0.8–10.4	5.5*	0.4–10.6

Covariates included age, gender, ethnicity (Hispanic vs. not Hispanic), completion of SS (did not complete, completed by 3 months, completed by 6 months), any missed interviews (any vs. none), site (POWC vs. RTC), living status (house/apartment/group home/halfway house; homeless/shelter/friend or family’s home; institution: prison/school/hospital or detox center), and time point (Initial, 3 months, and 6 months), as well as the interaction between intervention arm and time point

*p < 0.05

^a^Change scores are least squares mean differences

**Table 3:** Differences between PL-SS and CL-SS change scores over time with SE and CI, from least squares mean estimates for outcome measures in models adjusted for independent variables of interest

Measure	Change scores^a^ (3 months—initial)			Change scores^a^ (6 months—initial)		
	Difference	SE	95% CI	Difference	SE	95% CI
PCL total score	0.18	1.9	− 3.4 –3.8	− 0.24	1.9	− 4.0 to 3.5
Coping total score	1.1	2.4	− 3.5 to 4.3	− 0.07	24	− 4.8 to 4.7

The differences in change scores are the differences between Peer Led (PL) and Clinician Led (CL) mean change scores at the given time point from Initial, e.g., ΔPL, 3mo-BL—ΔCL, 3mo-BL. Negative values indicate that the PL score decreased at a faster rate than CL scores, while positive values indicate that PL scores increased at a faster rate. Covariates included age, gender, ethnicity (Hispanic vs. not Hispanic), completion of SS (did not complete, completed by 3 months, completed by 6 months), any missed interviews (any vs. none), site (POWC vs. RTC), living status (house/apartment/group home/halfway house; homeless/shelter/friend or family’s home; institution: prison/school/hospital or detox center), and time point (Initial, 3 months, and 6 months), as well as the interaction between intervention arm and time point

^a^Change scores are least squares mean differences

## Discussion

This was the first RCT of the effectiveness of SS delivered by peer providers. As hypothesized, effectiveness of peer providers was non-inferior to that of clinicians with peer providers achieving similar positive results to clinicians. The positive impact of peer-led SS on outcomes is consistent with the two non-experimental studies on peer-led SS conducted thus far (Najavits et al. 2014; OPTUM n.d.).

Clinical significance metrics were available for the PCL-C. On average, participants in both arms had an average 5-point decrease on the PCL-C between initial interview and 6 months [95% CI (− 7.2, − 2.7)] which is considered a reliable change despite this improvement not being large enough to be considered a clinically significant improvement (i.e., 10–20 point decrease). Many participants in the target population continued to be exposed to traumatic situations while participating in SS groups, including being homeless. Furthermore, improvements in PTSD symptoms may have been greater than what was reflected by the PCL-C since it has been shown to significantly underrate changes in symptom severity as a function of treatment and small changes following treatment have been reported to reflect larger clinical improvements (Forbes et al. 2001). The observed significant increase in the use of coping skills for all participants was also encouraging in that coping skills are critical for individuals with PTSD and/or substance use disorders. As described by Najavits (2002), individuals with histories of adverse childhood events, including childhood neglect, may not have learned coping skills in childhood or adolescence. Even if they were taught coping skills in childhood, they might not be readily available to adults with PTSD and/or substance use disorders because of changes in mood, ongoing trauma, altered states of mind due to long-term substance use, and overwhelming life circumstances. Furthermore, safe coping skills “replace the need for substances to manage emotions” (Najavits 2002).

The average number of completed SS sessions was moderate to low for both groups; 5.7 (median 4) for those in CL-SS and 6.4 (median 5) for those in PL-SS. In general, treatment completion is a challenge among those with substance use disorders and for those with lower education, addicted to heroin, and minorities, especially Hispanics (Agosti et al. 1996; Guerrero et al. 2013; Stark 1992). These characteristics describe the majority of the study population. The average number of completed SS sessions observed in this study is consistent with other studies that offered 12 sessions of SS to similar populations (Hien et al. 2009).

### Study Strengths and Limitations

In addition to the use of a RCT design in an usual care environment, other study strengths include a large ethnically diverse sample, multiple assessments of fidelity, and two follow-up time points, a limitation identified among the research on peer providers (Lloyd-Evans et al. 2014). Several methodological limitations need to be addressed. First, the validity of the findings may be threatened from selection bias in that 420 individuals were determined eligible and randomized, but only 69% consented to participate in the structured interviews. However, an examination of age, gender ethnicity/race, eligibility criteria, and history of trauma showed no significant differences between those who participated in the research (n = 291) and those who did not (n = 129). Similarly, three and 6-month follow-up data were not available for 69 participants who completed the initial interview. Second, we did not have true baseline data because initial interviews were conducted after participants completed their first SS group. This data collection sequence was implemented to achieve the required sample size for rigorous assessment of our endpoints. Our pilot study found that participants were unlikely to show up for their first SS group when they completed the surveys and received their compensation prior to the onset of treatment. Interviews were conducted as soon as possible with 47% of initial interviews being completed within 24 h of the first group. The mean number of days between the two events was 5 (SD = 11). Third, blinding was not possible because of the small community of the target population and the familiarity with the group facilitators. Previous research has noted the challenge of blinding among RCTs in rural communities (Kahan et al. 2014). Strategies were incorporated to minimize the potential for bias that may have resulted from lack of blinding, including the identical delivery of the intervention among study arms. Fourth, it is unknown whether the clinicians had histories of behavioral health problems. Previous studies have found the percent of counselors in recovery to range from 37 to 57% (Curtis and Eby 2010; Knudsen et al. 2006; McNulty et al. 2007). Fifth, the majority of data were self-report, which may be subject to recall bias and accurate disclosure. Research, however, has demonstrated the validity of self-report data among similar populations (Crisanti et al. 2003; Crisanti et al. 2005). Sixth, participants were receiving other behavioral health services simultaneously with SS that were not controlled for in the analytic models. While an examination of type and number of services revealed no significant differences between treatment arms, it would be misleading to attribute all significant improvements to SS.

These findings are generalizable to similar populations including Hispanics and underserved rural communities, with the understanding that several strategies were used for engagement and retention, and retention in services is related to outcomes. Generalizability may be limited by the small number of SS facilitators and by the study setting. Participants were recruited from a peer-operated recovery center and a residential treatment program for substance use disorders, and findings may not extend to populations receiving services from outpatient community-based behavioral health settings.

## Conclusions and Implications for Future Research

Overall, PL-SS and CL-SS resulted in similar changes in outcomes with confidence intervals that substantially overlapped. Moreover, as this study was designed to detect non-inferior effectiveness of PL-SS compared to CL-SS for PTSD symptom reduction, our results support our hypothesis. A confirmatory study of our findings is warranted given the high variability observed in our outcome measures. Considerable variation in change scores has been identified as a problem with the PCL-C (Forbes et al. 2001) and future research to better understand the variability observed in the treatment arms is needed. Future research is also needed to identify the relationship between the impact of unique qualities of peer providers (i.e., the mechanisms of change) and outcomes to increase our understanding of how and why peer-delivered services work. With the intent of controlling for all possible biases, this RCT did not account for or acknowledge these unique qualities. Research on the mechanisms of change with respect to peer providers, such as role modeling and therapeutic alliance for example, is warranted.

Results from the fidelity assessments and examination of outcomes suggest that peer providers can deliver SS and achieve success in doing so. While further research is needed to lend additional support to our findings, these results suggest that with training and supervision, peer providers can play a role in helping communities without enough licensed behavioral health clinicians meet the demand for trauma-specific treatment.

## Electronic supplementary material

Below is the link to the electronic supplementary material.

Supplementary material 1 (DOC 55 kb)
