# Supplementary material for: Effectiveness of Peer-Delivered Trauma Treatment in a Rural Community: A Randomized Non-inferiority Trial
Source: Community Ment Health J. 2019 Jul 23;55(7):1125–34. doi: 10.1007/s10597-019-00443-3 (PMC6744517; doi:10.1007/s10597-019-00443-3)
Supplement: Supplementary file 1 — Supplementary material 1 (DOC 55 kb) [file 10597_2019_443_MOESM1_ESM.doc]

**SS Topics**

The Seeking Safety manual provides information on all 25 treatment topics offered (Najavits, 2002). Twelve of the 25 topics were selected for our study. Topics were selected by consensus by patients and agencies responsible for implementation of SS and included:

(1) Safety,

(2) PTSD: Taking Back Your Power,

(3) Detaching from Emotional Pain (Grounding),

(4) When Substances Control You,

(5) Asking for Help,

(6) Red and Green Flags,

(7) Integrating the Split Self,

(8) Setting Boundaries in Relationships,

(9) Discovery,

(10) Coping with Triggers,

(11) Healthy Relationships, and

(12) Healing from Anger

**Average Fidelity Ratings By Group Facilitator for Format, Content and Process Sections on the Seeking Safety Fidelity Scale^a^**

|  | Average Ratings for Items Format Section | Average Ratings for Items Content Section | Average Ratings for Items Process Section |
| --- | --- | --- | --- |
| Female Peer Provider | 2.1 | 2.3 | 2.3 |
| Female Clinician | 2.4 | 2.5 | 2.5 |
| Male Peer Provider | 2.2 | 2.3 | 2.5 |
| Male Clinician | 2.5 | 2.6 | 2.7 |

^a^Items rated on a scale ranging from 0 to 3.

**Demographics of Randomized Individuals by Enrollment in Study**

|  | **Study Enrollment** | | | | | |  |
| --- | --- | --- | --- | --- | --- | --- | --- |
|  | Yes (N=291) | | No (N=129) | | Overall (N=420) | |  |
| **Characteristic** | N | % | N | % | N | % | **p-value^a^** |
| Age (Mean [Range]) | 35 [18 to 63] |  | 34 [18 to 64] |  | 34 [18 to 64] |  | 0.47 |
| Traumatic Events (Mean [Range]) | 7 [0 to 14] |  | 7 [0 to 14] |  | 7 [0 to 14] |  | 0.24 |
| Gender |  |  |  |  |  |  | 0.68 |
| Female | 129 | 44 | 60 | 47 | 189 | 45 |  |
| Male | 162 | 56 | 69 | 53 | 231 | 55 |  |
| Ethnicity |  |  |  |  |  |  | 0.12 |
| Hispanic | 231 | 82 | 110 | 88 | 341 | 84 |  |
| Non-Hispanic | 51 | 18 | 15 | 12 | 66 | 16 |  |
| Race |  |  |  |  |  |  | 0.69 |
| Caucasian | 78 | 58 | 37 | 65 | 115 | 60 |  |
| Native American | 27 | 20 | 9 | 16 | 36 | 19 |  |
| Asian | 1 | 1 | 0 | 0 | 1 | 1 |  |
| African American | 3 | 2 | 0 | 0 | 3 | 2 |  |
| Native Hawaiian/Pacific Islander | 7 | 5 | 1 | 2 | 8 | 4 |  |
| Multiracial or Other | 18 | 13 | 10 | 18 | 28 | 15 |  |
| Diagnosis |  |  |  |  |  |  | 0.69 |
| PTSD only | 26 | 9 | 14 | 11 | 40 | 10 |  |
| Substance Use Disorder only | 77 | 26 | 30 | 23 | 107 | 25 |  |
| PTSD and Substance Use Disorder | 188 | 65 | 85 | 66 | 273 | 5 |  |

^a^p-values were calculated using the Wilcoxon-Mann-Whitney test for age, a two sample *t* test for traumatic events, a Fisher’s exact test for race, and a chi-square test for gender, ethnicity, and diagnosis.
